# Supplementary material for: Following carpel tunnel release, what factors affect whether patients return to the same or different hand surgeon for a subsequent procedure?
Source: PLoS One. 2024 Oct 22;19(10):e0312159. doi: 10.1371/journal.pone.0312159 (PMC11495619; doi:10.1371/journal.pone.0312159)
Supplement: S1 Data — (DOCX) [file pone.0312159.s001.docx]

The data underlying the results presented in the current study are owned by the third party vendor PealDiver (URL: https://pearldiverinc.com/). Data can be queried using Bellwether software, which is part of the PearlDiver database. The authors did not have special permission or privileges outside of those granted via payment to the vendor.
